# Cell death: targeting ferroptosis in cancer

**DOI:** 10.1038/s41392-025-02534-y

**Published:** 2026-01-02

**Authors:** Hayley M. Sabol, Lorenzo Galluzzi, Lucia Borriello

**Affiliations:** 1 Department of Cancer and Cellular Biology, Lewis Katz School of Medicine, Temple University, Philadelphia, PA USA; 2 Cancer Signaling and Microenvironment Program, Fox Chase Cancer Center, Philadelphia, PA USA

**Keywords:** Metastasis, Cell biology, Lung cancer

In two recent studies published in *Nature*, Wu et al. and Palma et al. elegantly demonstrated that the endogenous ferroptosis suppressor AIF family member 2 (AIFM2, best known as FSP1) is a pharmacologically actionable vulnerability in lung carcinoma and metastatic melanoma.^1,2^ These findings formally validate the notion that activating ferroptosis might constitute a viable strategy to control developing tumors (which often exhibit defects in apoptotic signaling),^3^ potentially paving the way to the development of clinically viable ferroptosis inducers for cancer therapy.

Malignant cells often rely on the constitutive activation of oncogenic pathways for survival and proliferation, a scenario commonly known as “oncogene addiction”.^4^ In line with this notion, numerous clinically successful anticancer agents have been designed to specifically target bona fide oncoproteins, including (but not limited to) mutant epidermal growth factor receptor (EGFR), mutant KRAS proto-oncogene, GTPase (KRAS), and overexpressed BCL2.^4^ However, progressing tumors also experience considerable alterations in the intracellular and extracellular microenvironments that they can tolerate only through the hyperactivation of per se non-oncogenic mechanisms, such as the unfolded protein response (UPR) and autophagy.^4^ Since normal cells are generally not exposed to adverse microenvironmental conditions, such “non-oncogene addiction” also constitutes a viable target for cancer therapeutics, as exemplified by the clinical success of poly(ADP-ribose) polymerase (PARP) and cyclin-dependent kinase 4/6 (CDK4/6) inhibitors.^4^

One specific (and presumably common) form of non-oncogene addiction originates from the fact that malignant cells most often experience altered redox homeostasis, which calls for the upregulation of multiple antioxidant systems. This is particularly relevant because an uncontrolled increase in intracellular reactive oxygen species (ROS) may elicit catastrophic lipid peroxidation, culminating in cell death via ferroptosis.^3^ The recent findings of Palma et al. and Wu et al. extend the notion of non-oncogene addiction to the endogenous suppression of ferroptosis, opening interesting avenues for clinical development.

Wu et al. explored the impact of *Fsp1* or *Gpx4* (encoding yet another endogenous suppressor of ferroptosis) on the development of mouse KRAS^G12D^-driven lung adenocarcinomas (LUADs) in mice, and reported that deleting either gene considerably inhibited tumor progression along with the activation of ferroptosis in vivo. However, transcriptional analyses based on data from public repositories revealed that only FSP1 is upregulated in *KRAS*-mutant LUADs compared to normal pulmonary tissues. Moreover, in this patient series, FSP1 expression levels correlated with advanced disease stage and poor clinical outcome, suggesting that FSP1 may constitute a more relevant target than GPX4. Importantly, FSP1 deletion had no effect on the viability of KRAS^G12D^-driven LUAD cells in vitro, although it sensitized them to ferroptosis induction by the GPX4 inhibitor RSL3, a phenotype that was rescued by the pharmacological ferroptosis suppressor liproxstatin 1 (LIP1). Moreover, the oncosuppressive effects of *Fsp1* deletion persisted (1) in orthotopic LUAD models established in immunocompetent and immunodeficient hosts, and (2) in a large panel of LUAD cell lines irrespective of oncogenic driver, indicating that FSP1 supports in vivo LUAD progression in a cancer cell-intrinsic manner.^2^

Lipidomic profiling of *Fsp1*^*+/+*^ vs *Fsp1*^*−/−*^ orthotopic LUADs revealed that the lack of FSP1 is associated with the accumulation of oxidized and truncated phospholipids as well as with decreased reduced/oxidized coenzyme Q_9_ ratios, which are globally indicative of exacerbated lipid peroxidation and ferroptotic cell death. In line with this notion, the in vivo progression of *Fsp1*^*−/−*^ LUADs could be rescued by the overexpression of GPX4 as well as wild-type FSP1, but not an FSP1 variant that is unable to protect against lipid peroxidation in vitro. Similarly, *Fsp1*^*−/−*^ LUADs developed normally not only in the context of pharmacological ferroptosis inhibition via vitamin E supplementation and systemic LIP1 administration, but also upon the co-deletion of *Acsl4* (which encodes an acyl-CoA synthetase with ferroptosis-promoting activity).^2^

Finally, Wu and colleagues harnessed mouse *Fsp1*^*−/−*^ LUADs engineered to express human FSP1 to test the therapeutic effects of icFSP1 (a pharmacological inhibitor of human FSP1 recently developed by the Conrad team). Human FSP1 accelerated the in vivo progression of orthotopic *Fsp1*^*−/−*^ LUADs, a detrimental phenotype that could be fully rescued by icFSP1 administration unless an icFSP1-resistant variant of human FSP1 was employed. Moreover, icFSP1 potently restricted the growth of a human LUAD patient-derived xenograft established in immunodeficient mice, globally corroborating its cancer cell-specific, FSP1-targeted antineoplastic activity.^2^

Palma and collaborators investigated FSP1 dependency in metastatic melanoma as a follow-up on previous findings from the same team demonstrating that melanoma cells disseminated through the lymphatic system are resistant to ferroptosis via GPX4-independent mechanisms. To this end, the authors used melanoma B16-F10 cells with exquisite potential to colonize lymph nodes that were generated through serial in vivo selection by the Reticker-Flynn team, and found them to markedly upregulate FSP1 while exhibiting reduced protein levels of GPX4 and glutamate-cysteine ligase catalytic subunit (GCLC), which is required for the synthesis of glutathione, a key enzymatic co-factor for GPX4. This metabolic shift was mechanistically attributed to hypoxia, which is common in lymph nodes and results in accrued GPX4 degradation in the absence of transcriptional changes. Consistent with these findings, lymph node-colonizing melanoma cells demonstrated increased sensitivity to RSL3 compared with their parental counterparts, which correlated with decreased intracellular levels of GPX4-relevant metabolites, notably glutathione.^1^

In line with these observations, FSP1 was found to localize at lysosomes in melanoma cells colonizing lymph nodes to prevent lysosomal lipid peroxidation, via a mechanism that appeared to rely on FSP1 *N*-myristoylation but not lysosomal acidification (and hence proteolytic degradation). Accordingly, deleting *Fsp1* rendered lymph node-colonizing (but not parental) melanoma cells more sensitive to RSL3-driven ferroptosis in vitro, especially under hypoxic conditions. Finally, viFSP1 (but not icFSP1) as well as FSEN1 (another inhibitor of human FSP1 developed by the Olzmann team) actively controlled the progression of human SK-MEL5 melanomas established in the popliteal lymph node of NSG mice, a therapeutic effect that could not be improved by concomitant administration of the GCLC inhibitor *L*-buthionine sulfoximine (*L*-BSO). The specificity of viFSP1 was validated with wild-type vs. *Fsp1*^*−/−*^ lymph node-colonizing mouse melanomas established in immunocompetent, syngeneic mice.^1^

**Fig. 1:**
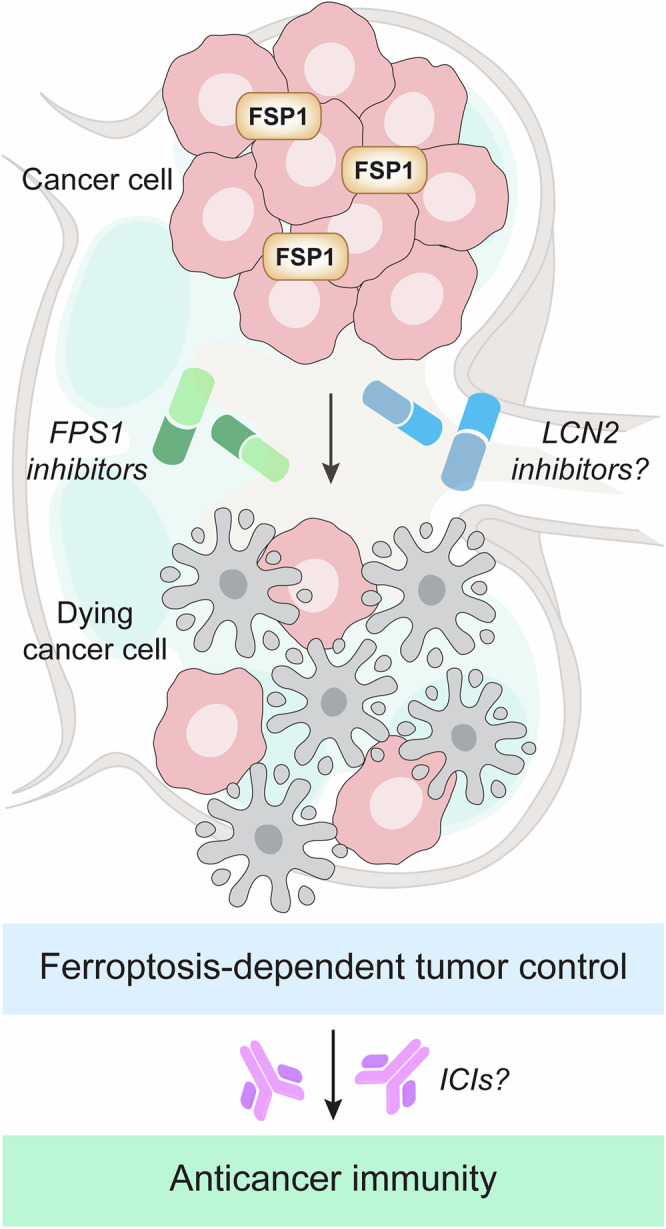
Ferroptosis-targeting strategies for cancer therapy. Both primary lung adenocarcinomas and melanomas that spread via the lymphatic route develop non-oncogene addiction for the antioxidant activities of the endogenous ferroptosis inhibitor AIF family member 2 (AIFM2, best known as FSP1). Accordingly, pharmacological inhibition of FSP1 demonstrated prominent therapeutic potential in preclinical models of these deadly neoplasms. Whether combining FSP1 inhibitors with lipocalin 2 (LCN2) blockers, which promote ferroptosis by increasing the intracellular availability of free iron, may result in superior therapeutic efficacy remains to be formally investigated. Similarly, the possibility that FSP1 inhibitors may positively interact with immune checkpoint inhibitors (ICIs) has not yet been experimentally assessed. The ability of ferroptosis to elicit adaptive anticancer immunity associated with an effector and memory phase (hence behaving as a bona fide immunogenic cell death variant) indeed appears to be highly context dependent, implying that a therapeutically meaningful interaction with ICI-based immunotherapy may be restricted to specific settings

In conclusion, recent work from Wu et al. and Palma et al. elegantly demonstrated that FSP1 is critical for the maintenance of in vivo redox homeostasis in lung carcinoma and metastatic melanoma. Thus, while these two studies focused on different oncological settings and unveiled context-specific configurations of the molecular machinery for ferroptosis, pharmacological FSP1 inhibitors stand out as promising tools for the development of novel anticancer regimens (Fig. 1). However, it remains to be formally investigated whether the activation of ferroptosis by FSP1 blockers may enable the orchestration of a tumor-specific immune response that can be further exacerbated by immune checkpoint inhibitors (ICIs). Indeed, the ability of ferroptosis to initiate the cascade of events required for cell death to be perceived as immunogenic, and hence trigger adaptive immunity coupled with effector and memory components, remains debated, and may considerably depend on context.^5^ Similarly, whether the therapeutic efficacy of FSP1 inhibitors may be ameliorated by emerging strategies that amplify ferroptotic signaling, such as interventions that increase the intracellular availability of free iron,^3^ is unclear. In this context, lipocalin 2 (LCN2) has recently emerged as a core regulator of intracellular iron availability and a powerful inhibitor of ferroptosis in aging cells, suggesting that blocking LCN2 may increase the therapeutic potential of FSP1 inhibitors. This possibility, however, has not yet been experimentally assessed. Finally, while FSP1 appears to be selectively harnessed by malignant cells for resisting ferroptosis, whether pharmacological FSP1 inhibitors may cause adverse events that could delay bench-to-bedside translation remains unexplored.

Despite these and other uncertainties, FSP1 has emerged as a pharmacologically actionable driver of non-oncogene addiction in cancer, potentially paving the way to novel therapeutic developments of clinical value.
